# Associations between clinical canine leishmaniosis and multiple vector-borne co-infections: a case-control serological study

**DOI:** 10.1186/s12917-019-2083-6

**Published:** 2019-09-18

**Authors:** Charalampos Attipa, Laia Solano-Gallego, Christian M. Leutenegger, Kostas Papasouliotis, Francesca Soutter, Jörg Balzer, Scott Carver, Jesse S. Buch, Séverine Tasker

**Affiliations:** 1 Molecular Diagnostic Unit, Diagnostic Laboratories, Bristol Veterinary School and Langford Vets, University of Bristol, Langford, UK; 2 Department of Pathobiology and Population Sciences, The Royal Veterinary College, University of London, Hatfield, Hertfordshire UK; 3 Cyvets Veterinary Center, Paphos, Cyprus; 4 Department of Clinical Infection, Microbiology and Immunology, Institute of Infection and Global Health, University of Liverpool, Liverpool, UK; 5 Departament de Medicina i Cirurgia Animals, Facultat de Veterinària, Universitat Autònoma de Barcelona, Barcelona, Spain; 6 IDEXX Laboratories, Inc., West Sacramento, CA USA; 7 Present Address: IDEXX Laboratories Ltd., Wetherby, UK; 8 IDEXX GmbH, Ludwigsburg, Germany; 9 Department of Biological Sciences, University of Tasmania, Hobart, TAS Australia; 10 IDEXX Laboratories, Inc., Westbrook, ME USA; 11 Bristol Veterinary School, University of Bristol, Langford, UK; 12 The Linnaeus Group, Shirley, UK

**Keywords:** Dog, *Leishmania infantum*, *Ehrlichia canis*, *Borrelia burgdorferi*, *Acanthocheilonema reconditum*, Vector-borne pathogen, Co-infection, Cyprus

## Abstract

**Background:**

Dogs that have clinical leishmaniosis (ClinL), caused by the parasite *Leishmania infantum*, are commonly co-infected with other pathogens, especially vector-borne pathogens (VBP). A recent PCR-based study found that ClinL dogs are more likely to be additionally infected with the rickettsial bacteria *Ehrlichia canis*. Further information on co-infections in ClinL cases with VBP, as assessed by serology, is required. The research described in this report determined if dogs with ClinL are at higher risk of exposure to VBP than healthy control dogs using a case-control serology study.

**Results:**

Of the 47 dogs with ClinL, anti-*E. canis*/ *Ehrlichia ewingii* antibodies were detected in 17 (36.2%), anti-*Anaplasma phagocytophilum*/*Anaplasma platys* antibodies in 5 (10.6%) and antigen for *Dirofilaria immitis* in 2 (4.3%). Of the 87 control dogs, anti-*E. canis*/*E*. *ewingii* antibodies were detected in 14 (16.1%) and anti-*A*. *phagocytophilum*/*A*. *platys* antibodies in 2 (2.3%). No anti-*Borrelia burgdorferi* antibody tests were positive. No statistical differences between the ClinL dogs and control dogs regarding lifestyle or use of ectoparasitic prevention, were identified. The ClinL was significantly associated with anti-*E. canis*/*E*. *ewingii* antibodies (odds ratio = 2.9, 95% confidence interval: 1.3–6.7, *P* = 0.010) compared to controls by both multivariable logistic regression and structural equation modelling.

**Conclusions:**

It was demonstrated that an increased risk for *E. canis*/*E*. *ewingii* seropositivity is present in dogs with ClinL compared to clinically healthy control dogs, despite similar ectoparasitic prevention use and lifestyle. Based on these findings it is suggested that dogs with ClinL should not only be tested for *E. canis* co-infection using PCR but also serologically for *E. canis*/*E*. *ewingii*.

## Background

Canine leishmaniosis (CanL) is a significant zoonotic disease in Mediterranean region and is caused by the kinetoplastid parasite *Leishmania infantum* that is transmitted by sand flies vectors belonging to the *Phlebotomus* genus [1]. Often vector-borne pathogens (VBP) such as *Anaplasma platys, Ehrlichia canis*, *Dirofilaria immitis*, *Hepatozoon canis* and *Babesia vogeli* concurrently infect dogs which have clinical leishmaniosis (ClinL) despite being transmitted by vectors different than these for *L. infantum* [2–4]. Such co-infections can result in an unexpected incubation time, atypical clinical sings, more severe clinicopathological abnormalities and worse prognosis for the dogs with CanL, compared with dogs that have CanL alone [2, 3, 5]. Furthermore, a recent PCR-based case-control study found that dogs with ClinL are in higher risk to be co-infected with *E. canis* compared to healthy matched controls [6]. Additional information on co-infections in ClinL cases with VBP, as assessed by serology in case-control studies, is required.

The aim of this study was to examine if dogs with ClinL are more likely to be exposed to *A. phagocytophilum*/*A. platys*, *B. burgdorferi* and *E. canis*/*Ehrlichia ewingii*, or infected for *D. immitis* than clinically healthy controls.

## Results

Serum was available in 47 dogs with ClinL and 87 dog controls that were included in this study. The age of these 134 dogs ranged from 1 up to 12 years (median 4 years, interquartile range 3 years) and 98 (73%) were pedigree including Cocker spaniel, Segugio Italiano, Beagle, German Shepherd and other breeds (Additional file 1).

**Fig. 1:**
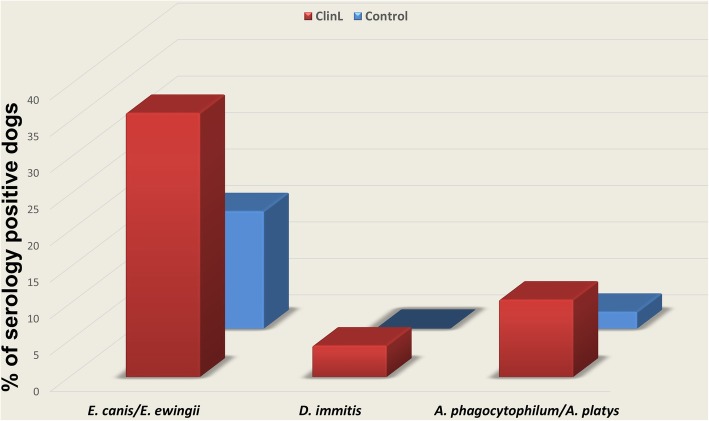
Comparison of VBP percentages detected by serology between dogs with ClinL (*n* = 47) and healthy control dogs (*n* = 87). *Abbreviations: VBP, vector-borne pathogen; ClinL, clinical leishmaniosis; E. canis, Ehrlichia canis; E. ewingii, Ehrlichia ewingii; D. immitis, Dirofilaria immitis; A. phagocytophilum, Anaplasma phagocytophilum; A. platys. Anaplasma platys*

In the ClinL group, anti-*A*. *phagocytophilum*/*A*. *platys* antibodies were detected in 5 (10.6%), anti-*E. canis*/*E*. *ewingii* antibodies in 17 (36.2%) and antigen for *D*. *immitis* in 2 (4.3%) dogs. Of the 87 control dogs, *anti-A*. *phagocytophilum*/*A*. *platys* antibodies were detected in 2 (2.3%) and anti-*E. canis*/*E*. *ewingii* antibodies in 14 (16.1%). No anti-*B*. *burgdorferi* antibody tests were positive (Fig. 1). Table 1 summarizes the demographic characteristics and the serology findings. The two dogs with *D*. *immitis* antigens underwent microfilaria PCR specification which was positive for *A. reconditum* and negative for *D*. *immitis* for both cases.

**Table 1:** Demographic characteristics of the study dog groups and serology results for the VBPs tested. All dogs tested negative for *Borrelia burgdorferi* antibodies

Characteristic	No. of cases ClinL (%) (*n* = 47)	No. of Control (%) (*n* = 87)
Age in years		
Median	3.0	4.1
Interquartile range	3.3	3.0
Sex		
Male	23 (49)	47 (54)
Female	24 (51)	40 (46)
Lifestyle		
Outdoors	34 (72)	65 (75)
Mainly indoors	13 (28)	22 (25)
Ectoparasitic prevention		
Used	16 (34)	36 (41)
Not used	31 (66)	51 (59)
Breed		
Pedigree	33 (70)	65 (75)
Crossbreed	14 (30)	22 (25)
*E. canis/E. ewingii*		
Positive	17 (36)	14 (16)
Negative	30 (64)	73 (84)
*A. phagocytophilum/A. platys*		
Positive	5 (10)	2 (2)
Negative	42 (90)	85 (98)
*D*. *immitis*		
Positive	2 (4)	0 (0)
Negative	45 (96)	87 (100)

*Abbreviations*: *VBP* Vector-borne pathogen, *ClinL* Clinical leishmaniosis, *E. canis Ehrlichia canis*, *E. ewingii Ehrlichia ewingii*, *D. immitis Dirofilaria immitis*, *A. phagocytophilum Anaplasma phagocytophilum*, *A. platys. Anaplasma platys*

ClinL was significantly associated with anti-*E. canis*/*E*. *ewingii* antibodies [odds ratio (OR) = 2.9, 95% confidence interval (CI): 1.3–6.7, *P* = 0.010], compared to healthy controls using multivariable logistic regression. The presence of *anti-A*. *phagocytophilum*/*A*. *platys* antibodies was initially associated significantly with ClinL compared to controls using univariable analysis (OR = 5.1, 95% CI: 0.9–27.2, *P* = 0.038) but this association was not maintained during multivariable logistic regression analysis. The numbers of *D*. *immitis* were very low hindering any further statistical analysis. Age, breed, sex, lifestyle, and use of ectoparasitic prevention were not statistically different between the ClinL and the control dogs.

**Fig. 2:**
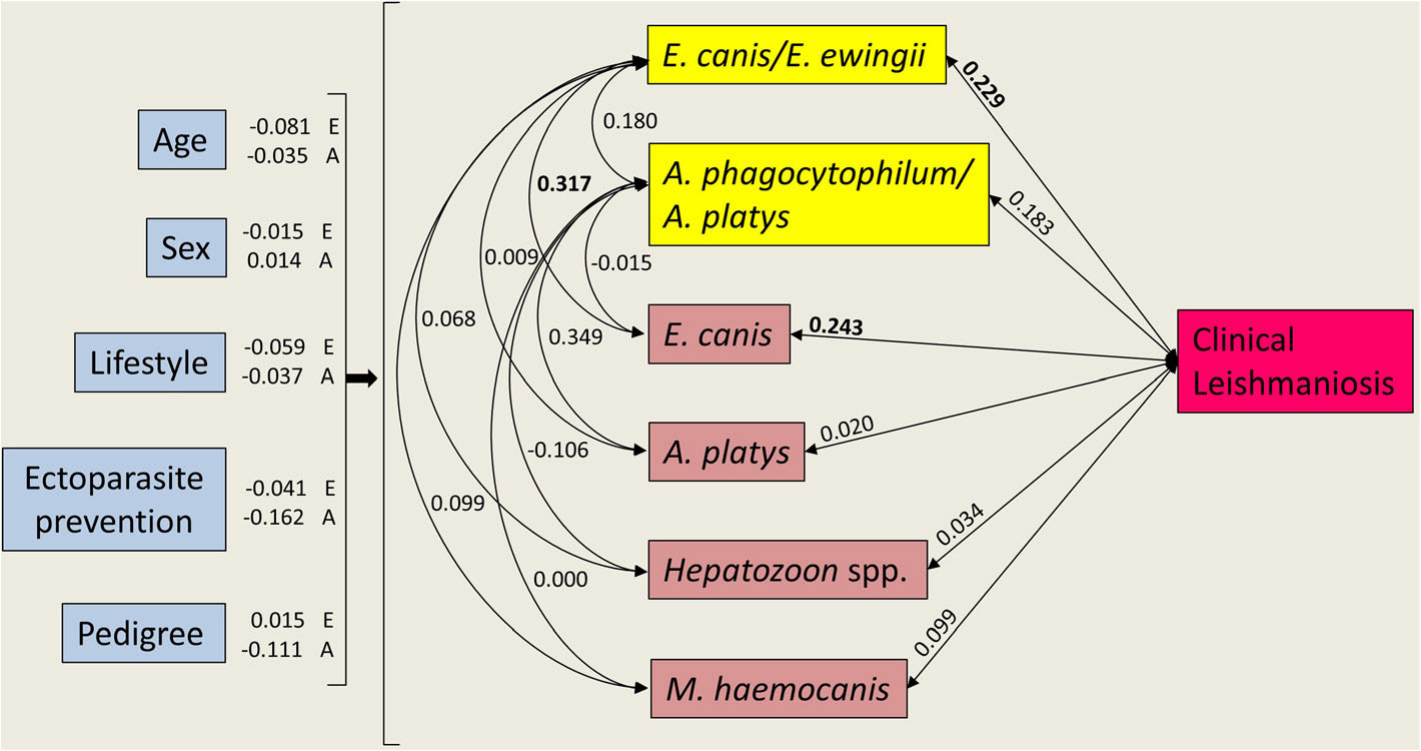
Structural equation model showing predictors of vector-borne serological exposure status (except ClinL), and pathogen covariance (including ClinL), in domestic dogs. Values represent standardised coefficients among variables. Single headed arrows represent directional/causal relationships and double headed arrows covariance relationships among pathogens. For image clarity the serological status is in yellow boxes and the coefficients of host characteristics predicting pathogens are listed next to each host characteristic. The covariances *E. canis*, *A. platys*, *Hepatozoon* spp. and *M. haemocanis* were PCR-based diagnosed. In all cases, except age, variables are binomial (0 or 1) with 1 equal to male, outside, ectoparasitic prevention use, pedigree and positive pathogen status. Standardised coefficients with significant relationships of *P* ≤ 0.05 (also see Table 2) are denoted in bold. *Abbreviations: ClinL, clinical leishmaniosis; E. canis, Ehrlichia canis; E. ewingii, Ehrlichia ewingii; A. phagocytophilum, Anaplasma phagocytophilum; A. platys. Anaplasma platys; M. haemocanis, Mycoplasma haemocanis*

Two associations were identified based on SEM (Fig. 2, Table 2). It was more likely for dogs with ClinL be *E. canis*/*E*. *ewingii* seropositive and dogs seropositive for *E. canis*/*E*. *ewingii* are more likely to have be infected with *E. canis* based on PCR. A trend was identified between dogs with ClinL and *A*. *phagocytophilum*/*A*. *platys* seropositive.

**Table 2:** Structural equation model statistical output showing host characteristics predicting serological exposure status for co-infecting pathogens (except ClinL), and the covariance among pathogens (including ClinL), in domestic dogs. The covariances *E. canis*, *A. platys*, *Hepatozoon* spp. and *M. haemocanis* were PCR based diagnosed. In all cases, except age, variables are binomial (0 or 1) with 1 equal to male, outside, ectoparasites controlled, pedigree and positive pathogen status

	Standardised coefficient/covariance	z-value	*P*-value
*E. canis/E. ewingii* serology			
Age	−0.081	−0.908	0.364
Sex	−0.015	− 0.143	0.886
Lifestyle	0.059	0.515	0.606
Ectoparasite prevention	−0.041	− 0.336	0.737
Pedigree	0.015	0.142	0.887
*A. phagocytophilum/A. platys* serology			
Age	−0.035	−0.415	0.678
Sex	0.014	0.103	0.918
Lifestyle	−0.037	− 0.363	0.717
Ectoparasite prevention	−0.162	−1.532	0.126
Pedigree	−0.111	−0.861	0.389
Covariances			
*E. canis/E. ewingii* ~~ ClinL	0.229	2.453	**0.014**
*A. phagocytophilum/A. platys* ~~ ClinL	0.183	1.654	0.098*
*E. canis/E. ewingii* ~~ *A. phagocytophilum/A. platys*	0.180	1.443	0.149
*E. canis/E. ewingii* ~~ *E. canis*	0.317	2.164	**0.030**
*E. canis/E. ewingii* ~~ *A. platys*	0.009	0.092	0.926
*E. canis/E. ewingii* ~~ *Hepatozoon* spp.	0.068	0.747	0.455
*E. canis/E. ewingii* ~~ *M. haemocanis*	0.099	1.015	0.310
*A. phagocytophilum/A. platys* ~~ *E. canis*	−0.015	−0.358	0.721
*A. phagocytophilum/A. platys* ~~ *A. platys*	0.349	1.290	0.197
*A. phagocytophilum/A. platys* ~~ *Hepatozoon* spp.	−0.106	−1.167	0.243
*A. phagocytophilum/A. platys* ~~ *M. haemocanis*	0.000	−0.005	0.996

Significant relationships (*P* < 0.05) denoted by bold font and trending relationships (*P* < 0.1) denoted by *

*Abbreviations*: *VBP* Vector-borne pathogen, *ClinL* Clinical leishmaniosis, *E. canis Ehrlichia canis*, *E. ewingii Ehrlichia ewingii*, *A. phagocytophilum Anaplasma phagocytophilum*, *A. platys. Anaplasma platys*, *M. haemocanis Mycoplasma haemocanis*

## Discussion

The findings from this serology study are in agreement with previous studies [3, 7] and further support the findings from the initial PCR based study, using a fairly similar cohort of samples, in which it was demonstrated that it is 12 times more likely for dogs with ClinL be co-infected with *E. canis* compared with healthy canine controls (CI: 1.5–106.0, *P* = 0.022) [6]. A previous 3-year longitudinal study, evaluating *E. canis* and *L*. *infantum* co-infection in naturally exposed dogs, found that *E. canis* infection preceded *L*. *infantum* infection in dogs with dual infections, thus suggesting that *E. canis* could contribute in the establishment of ClinL [7]. Interestingly, a recent study by Baxarias et al. [5] from Catalonia (Spain) found that dogs with ClinL were four times more likely to be seropositive for *Rickettsia conorii* and 14 times most likely to be seropositive for *A. phagocytophilum* compared with healthy controls, but they did not found an association between ClinL and *E. canis* seroreactivity. This discrepancy probably reflects the different prevalence of these pathogens in Cyprus and other Mediterranean areas in comparison to Catalonia.

The seroprevalence of the various VBP in this specific canine population of 134 dogs from the area of Paphos, Cyprus, revealed a strikingly high seroreactivity to *E. canis*/*E*. *ewingii* (23%) and *anti-A*. *phagocytophilum*/*A*. *platys* (13%) antibodies compared to other studies from Mediterranean countries using a similar in-house ELISA kit as the one utilised in this study [8–10]. If quantitative ELISA or IFAT with higher sensitivity, compared to the in-house kit, were used in this study, then seroprevalences of the VBP could have been even higher than these reported [11]. The area of Paphos, Cyprus, may be Lyme disease free as no anti-*B*. *burgdorferi* antibodies were detected in any of the dogs tested in this study, and the tick vectors that transmit this pathogen, including *Ixodes Ricinus*, have not yet been identified in Cyprus [12]. In two dogs (1%) antigens for *D*. *immitis* were detected but PCR failed to confirm this infection and instead an infection with *A. reconditum* was identified for both cases. These results may indicate that the dogs had dual infection with both *D*. *immitis* and *A. reconditum*, and the negative PCR for *D*. *immitis* was as a result of low level microfilaraemia. However, false positive *D*. *immitis* results cannot be ruled out entirely especially in light of a recent study from Cyprus in which, using a modified Knott’s testing for morphological identification of microfilariae in a total of 200 healthy dogs which did not receive any kind of heartworm prevention, only *A. reconditum* was identified in 9 dogs (4.5%) and no *D*. *immitis* was found [13].

## Conclusions

It was demonstrated that dogs with ClinL are three times more likely to be exposed to *E. canis*/*E. ewingii* than clinically healthy control dogs in Paphos, Cyprus. Furthermore, dogs from this area have a high seroreactivity to *E. canis*/*E*. *ewingii* and *A*. *phagocytophilum*/*A*. *platys* while they are *B*. *burgdorferi* free.

## Methods

### Study design, site and populations

The samples used for this serology study were collected under the frame of a previous case-control study design [6]. All samples were collected from canine clinical cases presented to a small animal veterinary hospital in Paphos, Cyprus from April 2013 until March 2014. That area was selected since there are high numbers of CanL [14] and various canine VBP have been reported [15, 16].

The exact recruiting criteria and the demographic characteristics recorded can be found in the previously published study [6]. Briefly, the dogs that had ClinL were naturally infected and matched with clinically healthy control dogs in-terms of breed, sex, age, living in the same geographical area as well as ideally lifestyle and ectoparasitic prevention use.

### Laboratory tests

Approximately 1–2 ml of surplus serum collected in plain tubes and stored at − 20 °C until laboratory processing at the Diagnostic Laboratories of the Royal Veterinary College, London, UK.

A commercial in-clinic patient-side SNAP® 4Dx® Plus test kit (IDEXX Laboratories, Inc., Westbrook, Maine, USA) was used for the simultaneous detection of antibodies against *E. canis*/*E. ewingii*, *A. phagocytophilum*/*A. platys*, and *B. burgdorferi*, as well as antigens for *D. immitis*, following manufacturer’s instructions. This ELISA kit utilises bi-directional flow of sample and automatic, sequential flow of wash solution and enzyme substrate. For *E. canis* it detects antibodies to the proteins p30 and p30–1, and for *E*. *ewingii* antibodies for p28 protein. For *A*. *phagocytophilum*/*A*. *platys* the assay detects antibodies against a peptide from the MSP2/p44 major surface protein and the C_6_ peptide is used for the detection of antibodies to a surface lipoprotein of *B*. *burgdorferi*. The assay detects antigens produced primarily from the uterus of female *D. immitis* (IDEXX Laboratories, Inc.).

Blood extracted DNA was submitted to IDEXX Laboratories, Ludwigsburg, Germany from all the cases that yielded positive antigens for *D. immitis* for further microfilaria specification using PCR specific assays for *D*. *immitis*, *Dirofilaria repens*, *Acanthocheilonema reconditum* and *Acanthocheilonema dracunculoides*. Additionally all samples underwent *L*. *infantum* serology [17], qPCRs for *Leishmania* spp. [18], *Babesia* spp. [19], “*Candidatus* Mycoplasma haematoparvum” and *Mycoplasma haemocanis* [20] as well as conventional PCR assays for *Ehrlichia/Anaplasma* spp. [21] and *Hepatozoon* spp. [22] under the framework of a previously published study [6].

### Data analysis

The sample size was previously calculated [6] and analyses were performed using SPSS for Windows (version 25.0; SPSS Inc., Chicago IL, USA). A univariable analysis was initially performed to see how each of the explanatory variables was associated with ClinL using Pearson’s Chi-square test for categorical explanatory variables (breed, sex, lifestyle, ectoparasitic prevention, positivity for *A. phagocytophilum*/*A. platys*, positivity for *B. burgdorferi*, positivity for *E. canis*/*E*. *ewingii*, and positivity for *D. immitis*), and the two-sample t-test or Mann-Whitney U test for continuous variables (age). Any variables that showed a trend towards significant association with ClinL (*P-*value < 0.1) were selected for entry into a multivariable logistic regression. A stepwise selection procedure was used to determine the final model (criteria for entry *P-*value ≤0.05 and for removal *P-*value > 0.1).

Additionally, structural equation modelling (SEM) was performed reflecting the hypothesise mechanisms that may be associated with ClinL and other VBP exposure statuses in dogs: (a) causal effects of host characteristics, and (b) pathogen interrelationships, using a method previously described [6].

## Supplementary information

**Additional file 1: Table S1.** Raw datasets with signalment and test results per case enrolled.

## Data Availability

The datasets supporting the conclusion of this article are included within the article and Additional file 1.

## Abbreviations

CI: Confidence interval
ClinL: Clinical leishmaniosis
ELISA: Enzyme-linked immunosorbent assay
IFAT: Immunofluorescence antibody test
OR: Odds ratio
qPCR: Quantitative polymerase chain reaction
SEM: Structural equation modelling
VBP: Vector-borne pathogen
